# Pre-Treatment Breast MRI Features and ADC Values as Predictors of Pathologic Complete Response in Breast Cancer: A Molecular Subtype-Based Analysis

**DOI:** 10.3390/diagnostics16060938

**Published:** 2026-03-22

**Authors:** Ela Kaplan, Hüseyin Alakus, Selcuk Kaplan

**Affiliations:** 1 Department of Radiology, Faculty of Medicine, Adiyaman University, Adiyaman 02040, Turkey; 2 Departmant of Surgery Oncology, Faculty of Medicine, Adiyaman University, Adiyaman 02040, Turkey; dr.alakus@hotmail.com; 3 Departmant of Gynecology and Obstetrics, Faculty of Medicine, Adiyaman University, Adiyaman 02040, Turkey; kaplan_2384@hotmail.com

**Keywords:** breast neoplasms, diffusion-weighted imaging, magnetic resonance imaging, neoadjuvant therapy

## Abstract

**Background/Objectives**: The role of pre-treatment breast magnetic resonance imaging (MRI) findings and apparent diffusion coefficient (ADC) values in predicting pathologic complete response (pCR) in breast cancer patients receiving neoadjuvant chemotherapy (NAC) has not yet been sufficiently clarified, especially in the context of molecular subtype differences. In this study, we questioned whether these imaging parameters were independent predictors of pCR. **Methods**: This study retrospectively explored MRI characteristics of 188 patients who underwent NAC from 2015 to 2023. The patients were divided into the pCR-positive and pCR-negative groups—the latter comprising patients with partial response (*n* = 61) and stable disease (*n* = 90)—and were classified into four molecular subtypes: Luminal A/B, HER2-enriched, and triple-negative breast cancer (TNBC). The MRI parameters included pre-chemotherapy T2-weighted signal characteristics, shape features, contrast kinetics, peritumoral edema, and ADC MIN/ADC MAX. Post-treatment ADC and ΔADC were the post-chemotherapy MRI parameters. Independent predictors were evaluated by logistic regression and discriminant performance by ROC analysis. **Results**: The overall pCR rate was 19.7%. In multivariate analysis, T2-weighted isointense signal (OR = 4.50), uniform tumor shape (OR = 12.83), HER2-enriched subtype (OR = 6.03), TNBC (OR = 5.15), ADC MIN (OR = 1.41), tumor size (OR = 1.28), and kinetic Type 3 pattern (OR = 3.21) were identified as independent predictors. Pre-treatment ADC MIN yielded an AUC of 0.724, while post-treatment ADC achieved 100% sensitivity and 96.7% specificity (AUC = 0.967). **Conclusions:** MRI morphology and ADC values may make a meaningful contribution to the prediction of pCR when evaluated in the context of molecular subtype. Post-treatment ADC demonstrated particularly strong discriminatory performance; however, external validation in multicenter cohorts is required before clinical implementation.

## 1. Introduction

Neoadjuvant chemotherapy (NAC) is a well-established approach in the treatment of early and locally advanced breast cancer. With the use of NAC, the behavior of the tumor in situ can be observed during chemotherapy [1]. Pathologic complete response (pCR) to NAC is the absence of any invasive tumour cells in the resected tissue and is a very powerful independent predictor of disease-free and overall survival [2,3]. Despite its strong prognostic value, the achievement of pCR rates varies widely among breast cancer molecular subtypes, which contributes to the high degree of inter-patient heterogeneity in chemotherapy response [1]. Notably, molecular subtype not only determines baseline pCR probability but also modulates the predictive value of imaging biomarkers, underscoring the need to interpret imaging findings within a subtype-specific framework. Furthermore, emerging subtype definitions—including HER2-low (IHC 1+ or IHC 2+/ISH-negative)—have been shown to represent biologically and clinically distinct entities with different pCR rates and survival profiles compared with HER2-zero tumors, adding further complexity to response prediction [4,5].

While magnetic resonance imaging (MRI) remains the imaging modality of choice for assessing the NAC response, the MRI accuracy has been reported to be influenced by tumor molecular features and MRI sequence parameters [1]. Diffusion-weighted imaging (DWI) and apparent diffusion coefficient (ADC) values are quantitative MRI-derived parameters that can theoretically reflect tumor cellularity, a known prognostic factor across a wide range of cancers. However, threshold values and reported accuracy vary considerably across studies, which has hampered the translation of imaging-based pCR prediction models into clinical practice [6,7]. Beyond imaging parameters, germline mutation status has also been identified as an independent determinant of pCR; BRCA1 carriers in particular have been shown to achieve higher pCR rates than BRCA2 carriers and non-carriers across molecular subtypes, with especially pronounced differences in the triple-negative subgroup [8]. The absence of BRCA mutation data in retrospective imaging cohorts therefore represents a potential confounding factor in pCR prediction analyses.

Current systematic reviews have shown that the sensitivity and specificity of MRI in predicting pCR exhibit significant differences according to molecular subtype; device field strength and methodological inconsistencies have been shown to directly affect the results [1,6]. However, studies in which pre-treatment MRI morphological features and ADC parameters are evaluated simultaneously in the context of molecular subtypes and systematically tested with independent predictor analyses remain underrepresented in the literature. Large multicenter studies such as the ACRIN 6698 trial and recent multicenter ADC analyses have advanced this field considerably [9,10]; however, the simultaneous integration of pre-treatment morphological features and diffusion parameters within subtype-stratified multivariate models is less consistently reported, particularly in datasets with minimized technical heterogeneity [6,7].

This study aims to determine the independent imaging predictors of pCR by simultaneously evaluating pre-treatment MRI morphological findings and ADC values within a subtype-stratified analytical framework, and to contribute to the existing literature by providing a more homogeneous methodological design than previously reported single-parameter analyses.

## 2. Materials and Methods

### 2.1. Study Population and Sample

The study was planned with a single-center retrospective cohort design. Patients who received neoadjuvant chemotherapy between 2015 and 2023 and underwent breast MRI examination before and after treatment were included. Images were obtained using a 1.5 T system (Gyroscan Intera, Philips Medical Systems, Best, The Netherlands). Excluding 20 cases in which response status was not clearly stated in the pathology report or imaging data were missing, the final sample reached 188 patients. The criteria for inclusion in the study were determined as follows: having been diagnosed with invasive breast cancer, having undergone MRI examination in accordance with the standard protocol before and after neoadjuvant treatment, and having the final pathology result available in the file.

pCR was defined as no residual invasive carcinoma detected in the primary tumor bed; the presence of ductal carcinoma in situ (DCIS) was allowed to be assessed within the definition of pCR, and this approach overlaps with the Miller–Payne grading system [11]. The pCR-negative group (pCR−) comprised patients with residual invasive disease following NAC, including those with partial response, stable disease, or disease progression. These subgroups were not analyzed separately, as the primary objective was to identify predictors of pathologic complete response, and the limited number of patients in each non-pCR subcategory precluded meaningful subgroup analysis.

Molecular subtype was determined based on ER, PR, HER2 and Ki-67 values. Accordingly, cases with ER(−)/PR(−)/HER2(−) were considered triple-negative breast cancer (TNBC) [12]. Patients with HER2 overexpression or amplification were classified as HER2-positive [12]. ER-positive and/or PR-positive and HER2-negative cases were included in the Luminal A-like or Luminal B-like subgroup according to Ki-67 proliferation index [12].

The Ki-67 proliferation index was used in the analysis for two separate purposes: the first was to determine the molecular subtype, and the second was to question the relationship of proliferation level with both prognosis and treatment response as an independent marker [12].

Clinical stage and lymph node status were not available for all patients in the retrospective dataset; therefore, these variables were not included in the regression analyses. This represents a limitation of the study design. Similarly, BRCA1/2 mutation data were not systematically collected as part of routine clinical care throughout the study period (2015–2023) and were therefore not available for inclusion in the analyses.

### 2.2. Imaging Protocol and Measurements

All MRI examinations were performed with 1.5 or 3 Tesla devices within the framework of the standard breast protocol. Examinations were performed using both 1.5T and 3T MRI systems. While standardized acquisition protocols were applied, systematic differences in ADC values between field strengths cannot be fully excluded. A sensitivity analysis comparing ADC values between field strengths was not feasible, as device-level data were not recorded per patient; this is acknowledged as a limitation in the limitations section. DWI was obtained using b = 0 and b = 800 s/mm^2^ values; ADC maps were automatically calculated by the system. In pre-treatment measurements, region of interest (ROI) was plotted on at least three different sections of the tumor and ADC MIN and ADC MAX values were recorded. T2-weighted (T2W) signal character, tumor shape, and margin features were interpreted according to ACR BI-RADS 2013 nomenclature [13]. Contrast kinetics were evaluated in Type 1 (persistent increase), Type 2 (plateau) and Type 3 (washout) categories based on Kuhl classification; the contrast rate was encoded at the binary level, slow and fast [14].

Non-mass enhancement (NME) was defined as a contrast enhancement pattern that followed anatomical structures without mass formation, and the presence or absence of the finding was noted. Peritumoral edema, skin thickening, nipple and chest wall involvement were also documented using the same method. Residual enhancement in post-treatment imaging was categorized into three distinct categories: no contrast uptake detected, homogeneous mass enhancement, and NME; the remaining tumor size was measured over the longest diameter in the axial plane. MRI response was classified as complete response (no residual enhancement), near-complete partial response, and partial/stable response. In the pairwise accuracy analysis, complete imaging response was compared with pathological complete response; near-complete partial response cases were maintained as a separate category and included in the false-positive group analysis [11].

The percentage change between post-ADC value and ΔADC was calculated by the formula [(Post-ADC − Pre-ADC MIN)/Pre-ADC MIN × 100]; ADC change has been previously shown to be associated with treatment response [15]. All imaging evaluations were performed independently by two experienced radiologists [14].

### 2.3. Intervention and Pathological Evaluation

All patients received standard-of-care neoadjuvant chemotherapy. Targeted therapy was given only to patients with HER2-enriched tumors [12]. The surgical specimens were evaluated by an expert pathologist and the pathological response was assessed by evaluating the resection specimens [11].

### 2.4. Statistical Analysis

All analyses were performed using SPSS 26.0 (IBM, Armonk, NY, USA). The distribution of continuous variables was assessed with the Shapiro–Wilk test; group comparisons were performed with the Mann–Whitney U test in the variables with non-normal distribution and the results were reported as median (Q1–Q3). Categorical variables were given in the form of numbers and percentages; the Fisher exact test was preferred in conditions where the expected number of cells fell below five, and the Chi-square test was preferred in other cases.

To reveal independent pCR predictors, univariate logistic regression was first applied; variables below the *p* < 0.10 limit were included in the multivariate binary logistic regression model. Molecular subtype was added to the model by polytomic dummy coding, with Luminal A as the reference category. The relationship between ADC MIN and ADC MAX was evaluated with the Spearman correlation coefficient; a moderate-to-high correlation was found between the two variables (r = 0.548, *p* < 0.001), and each was tested separately in univariate analysis; however, ADC MAX was excluded from the multivariate model due to collinearity with ADC MIN. ER, PR, and HER2 variables were strongly correlated with molecular subtype (Spearman r > 0.80) and were therefore excluded from the multivariate model; their effects were captured through subtype dummy coding. Variables with complete separation were excluded from the model instead of penalized regression. Model fit was checked with the Hosmer–Lemeshow test.

The performance of ADC values in pCR prediction was examined by receiver operating characteristic (ROC) analysis together with the effectiveness of post-ADC and ΔADC in residual tumor detection; optimal cut values were determined by Youden index and 95% confidence intervals were calculated by bootstrap method (1000 iterations). In subtype-based ROC analyses, Luminal A and Luminal B cases were combined under the name Luminal (A + B) to provide sufficient subgroup size. The significance of the difference between the two area under the curve (AUC) values was tested with the DeLong test. The agreement of MRI with the pathological result was reported as sensitivity, specificity, positive and negative predictive value and accuracy over a 2 × 2 accuracy table; 95% confidence intervals were expressed by the Wilson method at rates close to the limit values, and MRI-pathology agreement was expressed by Cohen’s Kappa coefficient. Since the study was exploratory, no statistical adjustment was made for multiple comparisons; the findings should be evaluated within this framework. The significance threshold was adopted as *p* < 0.05 in all analyses.

Internal validation was not performed due to the limited size of the pCR-positive subgroup (*n* = 37), which precluded reliable k-fold, leave-one-out, or bootstrap-based approaches. The predictive model should therefore be regarded as exploratory, and the reported performance metrics—particularly the post-treatment ADC AUC of 0.967—require confirmation in independent prospective multicenter cohorts before clinical translation.

## 3. Results

### 3.1. Patient Characteristics and pCR Distribution

The median age of the 188 patients in the study was 48.0 (42.0–56.0) years and the pCR rate was 19.7%. The median age was significantly lower in patients with pCR (45.0 vs. 52.0 years; *p* < 0.001). In terms of tumor size, the median long diameter was 24.0 mm in the pCR(+) group and 35.0 mm in the pCR(−) group; this difference was statistically significant (*p* < 0.001) and in line with the pattern that the probability of complete response is higher in the expected direction, i.e., in smaller tumors. The >20% Ki-67 rate was significantly higher in the pCR(+) group (64.9% vs. 38.4%; *p* = 0.005). There was an inverse relationship between hormonal receptor positivity and pCR; ER positivity was 77.5% in the pCR(−) group and only 29.7% in the pCR(+) group. HER2 positivity was significantly higher in the pCR(+) group (64.9% vs. 28.5%). The HER2-enriched group constituted the highest proportion among pCR(+) cases, at 64.9%. There was no significant difference between the two groups in terms of multifocality and multicentricity. The pCR(−) group comprised patients with residual invasive disease following NAC: 61 patients (40.4% of the pCR(−) group) demonstrated partial response and 90 patients (59.6%) had stable disease. Disease progression was not recorded as a distinct outcome category in this retrospective cohort. The distribution between partial response and stable disease subgroups did not differ significantly across molecular subtypes (*p* = 0.317). These subgroups were not subjected to separate predictor analysis, as the primary study objective was to identify imaging predictors of pathologic complete response, and the limited number of patients within each non-pCR subcategory precluded statistically reliable subgroup-level inference (Table 1).

### 3.2. Pre-Treatment MRI Features and ADC Values

ADC MIN values were found to be significantly lower in the pCR(+) group; the median values were 721.0 and 892.0 × 10^−3^ mm^2^/s, respectively (*p* < 0.001). A difference in the same direction and of similar magnitude was also observed for ADC MAX; the results were 834.0 in the pCR(+) group and 1052.0 × 10^−3^ mm^2^/s in the pCR(−) group (*p* < 0.001). These findings were consistent with the biological hypothesis that restriction of water diffusion and associated low ADC values are associated with higher chemotherapy response in high-cellularity tumors. The T2W signal character also exhibited a marked dissociation; the isointense signal was found to be disproportionately high in the pCR(+) group, while the hyperintense signal was dominant in the pCR(−) group. Heterogeneous internal enhancement was observed in all pCR(+) cases; homogeneous or rim contrast enhancement was not observed in any case in this group. In terms of kinetic curve type, the Type 3 (washout) pattern was significantly more common in the pCR(+) group (35.1% vs. 6.6%; *p* < 0.001); this pattern was consistent with expected biological behavior, particularly in the HER2-enriched and TNBC subgroups. The presence of peritumoral edema was also strongly associated with pCR; edema was detected in 70.3% of the pCR(+) group, while this rate decreased to 34.4% in the pCR(−) group. Notably, nipple involvement occurred only in pCR(−) cases. Marginal character and tumor shape also showed significant differences between the groups (Table 2).

### 3.3. Independent Predictors

Univariate and multivariate analyses of parameters associated with the probability of pathologic complete response were performed; variables with complete separation were excluded from the multivariate model. The following parameters reached significance in multivariate analysis: T2W isointense signal (OR = 4.50; 95% CI 1.60–12.64; *p* = 0.004), uniform tumor shape (OR = 12.83; 95% CI 1.64–100.22; *p* = 0.015), HER2-enriched subtype (OR = 6.03; 95% CI 2.06–17.64; *p* = 0.001), TNBC (OR = 5.15; 95% CI 1.16–22.81; *p* = 0.031), ADC MIN (OR = 1.41; 95% CI 1.14–1.74; *p* = 0.002 per 100 unit decrease), tumor size (OR = 1.28 per 10 mm; 95% CI 1.07–1.52; *p* = 0.006), and kinetic Type 3 pattern (OR = 3.21; 95% CI 1.12–9.18; *p* = 0.030). It should be noted that the uniform shape predictor demonstrated a notably wide confidence interval (95% CI 1.64–100.22), which likely reflects statistical instability attributable to the limited size of the pCR-positive subgroup (*n* = 37); this finding should therefore be interpreted with caution. Peritumoral edema remained only at the trend level in multivariate analysis (OR = 2.08; *p* = 0.154). ER, PR, and HER2 variables were not included in the model because they exhibited strong collinearity with subtype; ADC MAX was excluded due to collinearity with ADC MIN (r = 0.548). Although Ki-67 was significant in univariate analysis (*p* = 0.008), its independent contribution did not reach the significance threshold in the multivariate model due to variance overlap with subtype (Table 3).

### 3.4. ROC Analyses

Pre-treatment ADC MIN demonstrated moderate overall discriminatory performance for pCR prediction, with an AUC of 0.724 (95% CI: 0.641–0.807) at a cut-off value of ≤821 × 10^−3^ mm^2^/s, yielding a sensitivity of 75.7% and specificity of 68.2%. The AUC for ADC MAX was 0.751 (95% CI: 0.671–0.831). In subtype-specific analyses, the highest AUC for ADC MIN was observed in the HER2-enriched group (AUC = 0.812; 95% CI: 0.703–0.921). The AUC in the TNBC subgroup was 0.771, but should be interpreted with caution given the limited sample size (*n* = 19) and the wide confidence interval (0.561–0.982). The AUC in the Luminal (A + B) subgroup was the lowest at 0.582, consistent with the known biological heterogeneity of hormone receptor-positive disease and the limited utility of pre-treatment ADC as a standalone predictor in this subgroup.

Post-treatment ADC values demonstrated substantially better performance, with an AUC of 0.967 (95% CI: 0.936–0.993) at a cut-off value of >1513 × 10^−3^ mm^2^/s, achieving 100% sensitivity and 96.7% specificity. This value should be interpreted with caution, as the single-center design and the relatively small pCR-positive sample (*n* = 37) may increase the risk of overfitting; external validation in independent multicenter cohorts is required before this threshold can be applied in clinical practice. The AUC for ΔADC was 0.912, and the difference between the two parameters was not statistically significant with the DeLong test (*p* = 0.156), indicating that post-ADC and ΔADC may serve as interchangeable parameters in clinical assessment (Table 4).

### 3.5. Post-Treatment MRI Accuracy

**Figure 1:**
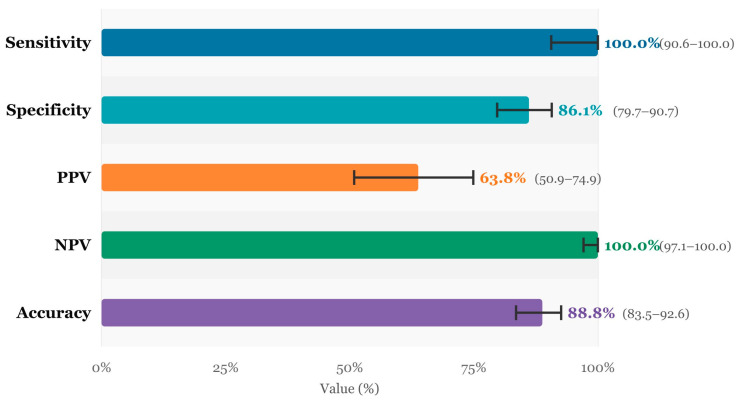
Post-Treatment MRI Diagnostic Performance Metrics.

**Figure 2:**
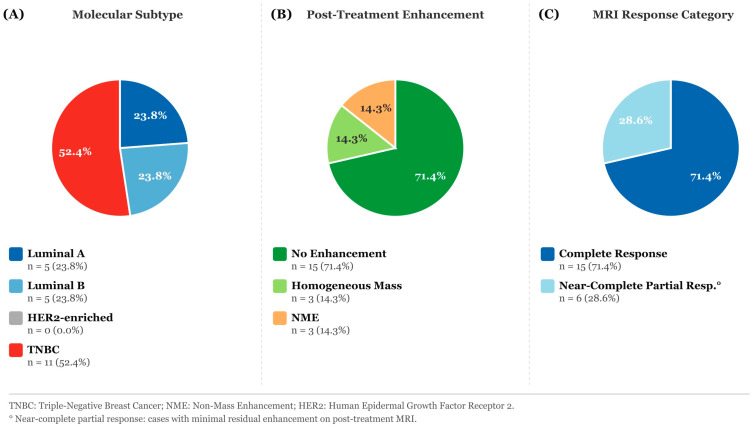
Characteristics of the False-Positive Group (*n* = 21). (**A**) Molecular Subtype—Luminal A, Luminal B, HER2-enriched (0%), TNBC; (**B**) Post-Treatment Enhancement—No enhancement, Homogeneous mass, NME; (**C**) MRI Response Category—Complete response, Near-complete partial response.

Post-ADC values were significantly higher in the pCR(+) patients compared to the pCR(−) group (median 1712.0 vs. 1115.0 × 10^−3^ mm^2^/s; *p* < 0.001). The ΔADC increase was also striking; the median value reached 138.2% in the pCR(+) group, compared with 30.5% in the pCR(−) group (*p* < 0.001). These high ΔADC values form a biologically consistent framework, given that the pre-treatment ADC MIN median started from a lower baseline of 721 × 10^−3^ mm^2^/s in the pCR(+) group. When the performance of MRI in detecting residual tumors was examined, the sensitivity was 100.0% and the specificity was 86.1%. There were 21 false-positive cases, of which more than half belonged to the TNBC subtype (52.4%); this suggests that post-treatment loss of contrast enhancement in TNBC patients does not always reflect pathologic complete response. No false-negative cases were encountered. The agreement between MRI and pathology was strong, with a Cohen’s Kappa of 0.709 (Table 5, Figure 1 and Figure 2).

## 4. Discussion

In this study, the roles of pre-treatment MRI findings and ADC values in predicting pCR were examined within a molecular subtype-based framework. We explored how imaging parameters related to pathologic complete response (pCR) and identified molecular subtype as a key source of heterogeneity in this association. The study results demonstrated that the association between imaging parameters and pCR was not uniform across all patients and that differences in biology between subtypes explained some of the heterogeneity. MRI features and ADC values were predictive of pCR in this patient population. Of the imaging parameters evaluated, the post-therapy ADC values provided the best performance for the identification of residual disease. As a retrospective single-center study, our findings cannot be generalized to broader clinical practice without further external confirmation. The imaging trends we observed are consistent with the results of large multicenter studies, particularly the ACRIN 6698 trial and the multicenter ADC analysis by Surov et al. (2022) [8,9], and should be interpreted in light of those datasets rather than in isolation.

The overall pCR rate in our cohort was found to be 19.7%; this rate is very consistent with the 20.9–21.1% values reported in meta-analyses based on real-world data [3,16]. The HER2-enriched group was dominant among pCR(+) cases, accounting for 64.9% of this group. The pCR rate in HER2-positive breast cancer has been reported to vary between 36–44% [3,16] and this rate is likely to be improved with the addition of HER2-targeted therapies. Our data are therefore biologically plausible. In TNBC, the pCR rate was approximately 26%, slightly below the 31–33% band in the literature [3,16]; this difference is most likely due to the small size of the subgroup (*n* = 19) and should be interpreted with caution. Furthermore, the absence of BRCA1/2 germline mutation data in our cohort may partly account for this lower-than-expected TNBC pCR rate. Myers et al. (2024) demonstrated in a large cohort of 1426 patients that BRCA1 carriers achieved pCR in 42% of cases—substantially higher than the 26% observed in non-carriers—and that this advantage was particularly pronounced in the triple-negative subgroup (79% vs. 45%; *p* < 0.001) [8]. The retrospective design of the present study precluded systematic BRCA mutation data collection throughout the 2015–2023 study period, which represents a meaningful limitation in the interpretation of TNBC-specific pCR estimates. Median age was 45.0 years in the pCR(+) group versus 52.0 years in the pCR(−) group. Lai et al. (2025) did not find any age difference between the two groups in another cohort of patients (52.4 vs. 53.6 years, *p* = 0.35) [17], which therefore suggests that the role of age in predicting pCR remains to be defined according to the subtypes. The smaller tumor size in the pCR(+) group (24.0 vs. 35.0 mm) was consistent with the findings of Lai et al. (40.4 vs. 47.9 mm, *p* = 0.01) [17]. The high Ki-67 values (>20%) seen in the pCR(+) group are also in line with expectations, given that Gass et al. (2018) reported that a pathologic complete response (pCR) was achieved in a higher percentage of patients in a TNBC cohort with a Ki-67 of ≥36% versus those with low Ki-67 (51.1% vs. 29.6%) [18]. The inverse relationship of ER positivity with pCR and the positive contribution of HER2 positivity are consistently supported by all reference studies [16,17]. Although it is an expected finding that multifocality and multicentricity do not differ between the two groups, large-scale studies directly addressing the effect of these variables on pCR are still very limited. It should also be noted that the pCR(−) group in our cohort was heterogeneous, comprising 61 patients with partial response and 90 patients with stable disease; the absence of disease progression as a distinct outcome category reflects the institutional treatment protocol and the retrospective nature of the study design, and should be considered when interpreting subtype-specific response patterns.

In terms of pre-treatment MRI morphology, T2W isointense signal (OR = 4.50) and uniform tumor shape (OR = 12.83) stood out as independent predictors; however, it should not be ignored that the second finding has an extremely wide confidence interval (95% CI 1.64–100.22) and reflects statistical instability due to the small pCR(+) sample (*n* = 37). Hu et al. (2024) and Shi et al. (2025) reported that uniform well-defined margins were higher in the pCR(+) group, but there were no comparable studies to confirm this [19,20]. NME and rim enhancement showed no association with imaging-pathology mismatch in our cohort. In contrast, Hu et al. (2024) reported that irregular shapes and the presence of rim enhancement (OR = 4.261) and NME increased the imaging-pathology mismatch [19]. Shi et al. (2025) showed that mass and NME mixed morphology had a stronger association with pCR [20]. These results were most likely due to differences between the patient populations and different tumor morphology definitions. The washout kinetic pattern (Type 3) was significantly higher in the pCR(+) group (35.1% vs. 6.6%) as reported in the literature. Hu et al. (2024) reported that the washout pattern increased the risk of imaging-pathology mismatch [19], and Shi et al. (2025) reported that the plateau/increasing time-intensity curve (TIC) type was inversely associated with pCR in the post-NAC period (OR = 0.033) [20]. Peritumoral edema provided strong univariate predictors but lost significance in multivariate analysis. Lee et al. (2024) failed to confirm peritumoral edema as an independent predictor in the TNBC series [21]. In our dataset, heterogeneous internal contrast was present in all cases in the pCR(+) group, but no case had a rim or homogeneous pattern. Therefore, we could not include it in our statistical models, although we believe that it would be worth investigating in more detail and would probably require a different study design. The contribution of pre-treatment morphological parameters to the estimation of pCR is likely to be limited. Cao et al. (2023) showed that none of the parameters obtained by pre-treatment DCE-MRI could distinguish between pCR and non-pCR [22]. In contrast, the parameters obtained from functional images in the early stages of treatment will probably give a better estimation of response.

Pre-treatment ADC MIN values were lower in the pCR(+) group (0.721 × 10^−3^ mm^2^/s) compared with the pCR(−) group (0.892 × 10^−3^ mm^2^/s, *p* < 0.001). The pre-treatment ADC MIN value was retained in the final model as an independent predictor (OR = 1.41, 95% CI 1.14–1.74), supporting the diffusion restriction hypothesis that highly dense cellular environments lead to a better NAC response. The ACRIN 6698 multicenter trial demonstrated that ADC change during treatment outperforms pre-treatment ADC alone in predicting pCR [10], a finding consistent with our ΔADC results (AUC = 0.912). Surov et al. (2022) similarly observed lower pre-treatment ADC values in the pCR(+) group in a multicenter cohort but reported limited clinical value due to overlapping distributions across subtypes [9]—a pattern directly reflected in our luminal subgroup AUC of 0.582, which underscores the biological heterogeneity of hormone receptor-positive disease and the limited utility of pre-treatment ADC as a standalone predictor in this subgroup. Partridge et al. (2018) and Liang et al. (2022) found that the pre-treatment ADC value is not enough to predict pCR alone, and the ADC change during treatment has the highest predictive value [10,23]. This is in agreement with our moderate pre-treatment AUC value of 0.724. The highest discriminant power of ADC in the HER2-enriched group was observed with AUC = 0.812, which was in agreement with the finding of Surov et al. (2022) in which the highest OR value (multivariate OR = 76.98) was obtained in this subtype [9]. Although the AUC was calculated as 0.771 in TNBC, the small size of the subgroup (*n* = 19) and the width of the confidence interval (0.561–0.982) limited this finding; Surov et al. (2022) also reported significant but widely spaced OR values in TNBC, with an analogous interpretation challenge [9]. The AUC in the luminal A + B group decreased to 0.582. Meyer et al. (2022) also found that the pre-treatment ADC values between molecular subtypes are statistically indistinguishable, and this again highlights the need to standardize the studies due to the very high degree of inter-study heterogeneity (I^2^ = 95–98%) [24]. Note that different technical parameters, such as b-values (800 vs. 1000 s/mm^2^) and magnetic field strength (1.5T vs. 3T), can affect the optimal ADC cut-off values, and therefore limit comparison between studies [9,24].

After treatment, the MRI sensitivity to assess pCR in our cohort was 100% and the specificity was 86.1%. Gampenrieder et al. (2019) reported an MRI sensitivity of 75% and specificity of 67% in a similar cohort and a decrease in PPV to 33% in hormone receptor (HR) positive tumors [25]. The Kappa value of 0.709 is considered a good level of agreement in the Landis-Koch classification. Other studies reported poor agreement between radiologic complete response (rCR) and pCR, such as a Kappa value of −0.1 reported by Gampenrieder et al. (2019) [25]. The 21 false-positive cases in our cohort were predominantly TNBC (52.4%), and 71.4% showed no enhancement on post-treatment MRI, indicating that the contrast characteristics of desmoplastic fibrosis and inflammatory changes caused by chemotherapy in TNBC tissue may be quite different from those of residual tumor cells. Zhang et al. (2020), on the other hand, reported that PPV reached 100% in TNBC and suggested that rCR may be a reliable surrogate for pCR in this subgroup [26]; however, it should be taken into account that the patient profile in their study and the definition of rCR applied are different from ours. Our post-ADC AUC value was 0.967. While this figure is higher than the 0.906–0.947 range reported by multicenter deep learning studies [27], we do not consider this a straightforward indicator of superior performance. Given that our cohort is limited to a single center and the pCR-positive subgroup contained only 37 patients, overfitting remains a real concern, and external validation is required before clinical application. The AUC of ΔADC was 0.912, which was smaller than that of post-ADC but non-significant in the DeLong test (*p* = 0.156), indicating that ΔADC and post-ADC could be used as alternatives to each other in clinical practice. The AUC values reported by Partridge et al. (2018) [10] for ΔADC (0.60–0.76) are significantly lower than our results [19]; these differences may reflect the time point of measurement (mid-treatment vs. end-of-treatment) or differences in study design.

The current study incorporated only pre- and post-treatment MRI; mid-treatment imaging was not performed. This is a recognized limitation, as emerging evidence supports the value of intra-treatment DWI for real-time treatment adaptation. Early ADC change has been shown to outperform pre-treatment ADC alone in pCR prediction [10], and longitudinal DCE-MRI changes have similarly demonstrated predictive value before treatment completion [22]. Future prospective studies should incorporate mid-treatment DWI time points, which would be of particular clinical relevance in subtypes with lower baseline pCR rates such as Luminal A, where early identification of non-responding patients could enable timely treatment modification.

A further limitation of the molecular classification applied in this study is the absence of a distinct HER2-low category. Under St. Gallen 2013 criteria [12], tumors with HER2 IHC 2+/ISH-negative or IHC 1+ expression were subsumed within the Luminal or TNBC groups depending on hormone receptor status. However, HER2-low tumors have been shown to exhibit distinct pCR rates and survival profiles compared with HER2-zero tumors [4,5], and the growing availability of antibody-drug conjugates such as trastuzumab deruxtecan makes this distinction increasingly clinically relevant. Whether HER2-low status influences ADC values or MRI morphology remains an open question that standard four-subtype classification cannot currently address. Similarly, ER-low positivity (ER 1–10%) may behave more similarly to ER-negative tumors in terms of chemotherapy sensitivity and pCR probability. Future studies should incorporate HER2-low and ER-low categories as separate analytical strata to better characterize their predictive imaging signatures.

The main limitation of this study is that it is a single-center retrospective study. This can lead to selection bias and restrict the generalizability of the results. A number of subgroups, including TNBC (*n* = 19), are based on relatively small datasets and should be interpreted with caution. The differing magnetic field strengths (1.5T and 3T) may also cause some systematic variation in ADC values; a sensitivity analysis comparing ADC values between field strengths was not feasible as device data were not recorded for individual patients, which constitutes a methodological limitation. The absence of lymph node status, clinical staging data, and BRCA1/2 mutation information—none of which were consistently available in the retrospective dataset—further limits the completeness of the predictor model and the interpretability of subtype-specific findings. It will be necessary to confirm these findings in a larger, multicenter, prospective trial with uniform MRI parameters. Integrated models combining radiomics and clinical markers should also be tested.

## 5. Conclusions

This study showed that pre-treatment MRI morphological features and ADC measurements were significant imaging indicators for pCR in breast cancer; however, this relationship cannot be evaluated independently of molecular subtype. Post-treatment ADC values demonstrated strong discriminatory performance for residual tumor detection in this single-center cohort; however, the high AUC value of 0.967 should be interpreted with caution given the risk of overfitting inherent to the retrospective single-center design, and these results require external validation in multicenter prospective studies before clinical translation. In conclusion, the interpretation of quantitative diffusion imaging in conjunction with morphological parameters and in the context of molecular subtype may make a clinically meaningful contribution to the radiological evaluation of NAC response. Future studies should prospectively validate these findings in multicenter cohorts with uniform MRI protocols and incorporate mid-treatment DWI time points to enable earlier identification of non-responding patients. HER2-low and ER-low subgroups and the influence of BRCA1/2 mutation status on imaging-based pCR prediction should also be addressed as separate analytical strata.

## Figures and Tables

**Table 1:** Clinical and Demographic Characteristics of Patients (by pCR Groups).

Variable	Entire Cohort (*n* = 188)	pCR(−) (*n* = 151)	pCR(+) (*n* = 37)	*p*
Age (years) ^a^	48.0 (42.0–56.0)	52.0 (44.0–58.0)	45.0 (34.0–47.0)	<0.001
Tumor long diameter (mm) ^a^	32.0 (25.0–41.0)	35.0 (28.0–42.0)	24.0 (18.0–31.0)	<0.001
Ki-67 ^b^				0.005
≤20%	106 (56.4%)	93 (61.6%)	13 (35.1%)	
>20%	82 (43.6%)	58 (38.4%)	24 (64.9%)	
ER status ^c^				<0.001
Positive	128 (68.1%)	117 (77.5%)	11 (29.7%)	
Negative	60 (31.9%)	34 (22.5%)	26 (70.3%)	
PR status ^c^				0.002
Positive	101 (53.7%)	90 (59.6%)	11 (29.7%)	
Negative	87 (46.3%)	61 (40.4%)	26 (70.3%)	
HER2 status ^c^				<0.001
Positive	67 (35.6%)	43 (28.5%)	24 (64.9%)	
Negative	121 (64.4%)	108 (71.5%)	13 (35.1%)	
Molecular subtype ^d^				<0.001
Luminal A	76 (40.4%)	71 (47.0%)	5 (13.5%)	
Luminal B	26 (13.8%)	23 (15.2%)	3 (8.1%)	
HER2-enriched	67 (35.6%)	43 (28.5%)	24 (64.9%)	
TNBC	19 (10.1%)	14 (9.3%)	5 (13.5%)	
Multifocal ^c^				0.804
Present	30 (16.0%)	25 (16.6%)	5 (13.5%)	
None	158 (84.0%)	126 (83.4%)	32 (86.5%)	
Multicentricity ^c^				1.000
Present	39 (20.7%)	32 (21.2%)	7 (18.9%)	
None	149 (79.3%)	119 (78.8%)	30 (81.1%)	
pCR(−) response subcategory ^e^				—
Partial response	61 (32.4%)	61 (40.4%)	— ^g^	— ^g^
Stable disease	90 (47.9%)	90 (59.6%)	— ^g^	— ^g^

^a^ Median (Q1–Q3); Mann–Whitney U test. ^b^ Chi-square test. ^c^ Fisher’s exact test. ^d^ Subtype derivation: TNBC = ER(−)/PR(−)/HER2(−); HER2-enriched = HER2(+); Luminal A = ER/PR(+), HER2(−), Ki-67 ≤ 20%; Luminal B = ER/PR(+), HER2(−), Ki-67 > 20%. pCR rate: 19.7% (37/188). ^e^ Subcategory data apply exclusively to the pCR(−) group (*n* = 151). Proportions shown for the entire cohort column reflect the share of the total 188-patient sample. Partial response: *n* = 61 (40.4% of pCR(−)); Stable disease: *n* = 90 (59.6% of pCR(−)). Disease progression was not recorded as a distinct outcome in this retrospective cohort. The distribution of response subcategories did not differ significantly across molecular subtypes (Chi-square test, *p* = 0.317). ^g^ Statistical comparison between pCR(−) subcategories and the pCR(+) group was not performed, as pCR(+) patients by definition had no residual invasive disease and therefore cannot be classified within the partial response or stable disease categories.

**Table 2:** Pre-treatment MRI Features and ADC Values According to Pathological Complete Response Status.

Variable	pCR(−) (*n* = 151)	pCR(+) (*n* = 37)	*p*
ADC MIN (×10^−3^ mm^2^/s) ^a^	892.0 (845.0–951.0)	721.0 (654.0–798.0)	<0.001
ADC MAX (×10^−3^ mm^2^/s) ^a^	1052.0 (981.0–1125.0)	834.0 (756.0–912.0)	<0.001
T2W signal ^b^			<0.001
Hypointense	80 (53.0%)	21 (56.8%)	
Isointense	21 (13.9%)	13 (35.1%)	
Hyperintense	50 (33.1%)	3 (8.1%)	
Tumor shape ^c^			0.027
Smooth	5 (3.3%)	5 (13.5%)	
Irregular	146 (96.7%)	32 (86.5%)	
Tumor margin ^c^			0.005
Irregular	57 (37.7%)	24 (64.9%)	
Spiculated	94 (62.3%)	13 (35.1%)	
Internal contrast ^c^			<0.001
Homogeneous	36 (23.8%)	0 (0.0%)	
Heterogeneous	110 (72.8%)	37 (100.0%)	
Rim	5 (3.3%)	0 (0.0%)	
Kinetic curve type ^b^			<0.001
Type 1	58 (38.4%)	8 (21.7%)	
Type 2	83 (55.0%)	16 (43.2%)	
Type 3	10 (6.6%)	13 (35.1%)	
Contrast rate ^c^			0.253
Slow	58 (38.4%)	10 (27.0%)	
Fast	93 (61.6%)	27 (73.0%)	
NME presence ^c^			0.156
Present	9 (6.0%)	5 (13.5%)	
None	142 (94.0%)	32 (86.5%)	
Peritumoral edema ^c^			<0.001
Present	52 (34.4%)	26 (70.3%)	
None	99 (65.6%)	11 (29.7%)	
Skin thickening ^c^			0.123
Present	30 (19.9%)	12 (32.4%)	
None	121 (80.1%)	25 (67.6%)	
Nipple involvement ^c^			0.009
Present	22 (14.6%)	0 (0.0%)	
None	129 (85.4%)	37 (100.0%)	
Chest wall involvement ^c^			0.585
Present	5 (3.3%)	0 (0.0%)	
None	146 (96.7%)	37 (100.0%)	

^a^ Median (Q1–Q3); Mann–Whitney U test. ^b^ Chi-square test. ^c^ Fisher’s exact test. Tumor shape and margin were determined according to ACR BI-RADS 2013 lexicon; kinetic curve type was evaluated according to Kuhl classification. NME: non-mass enhancement; ADC: apparent diffusion coefficient; pCR: pathological complete response.

**Table 3:** Univariate and Multivariate Logistic Regression Analysis for Predictors of Pathological Complete Response.

Variable	Univariate OR (95% CI)	*p*	Multivariate OR (95% CI)	*p*
Internal contrast (heterogeneous vs. other) ^e^	Complete separation ^e^	<0.001	Not included ^e^	—
Nipple involvement (present vs. absent) ^e^	Complete separation ^e^	0.009	Not included ^e^	—
T2W signal (isointense vs. hypointense)	3.35 (1.48–7.59)	0.004	4.50 (1.60–12.64)	0.004
T2W signal (hyperintense vs. hypointense)	0.18 (0.05–0.61)	0.006	0.23 (0.06–0.91)	0.036
Tumor shape (smooth vs. irregular)	4.56 (1.25–16.70)	0.022	12.83 (1.64–100.22)	0.015
Tumor margin (irregular vs. spiculate) ^h^	1.66 (0.79–3.49)	0.183	Not included ^h^	—
Peritumoral edema (present vs. absent)	4.50 (2.06–9.82)	<0.001	2.08 (0.76–5.69)	0.154
Kinetic curve Type 3 (vs. Type 1 + 2)	7.56 (3.12–18.33)	<0.001	3.21 (1.12–9.18)	0.030
Molecular subtype (HER2-enriched vs. Luminal A) ^j^	7.93 (2.81–22.32)	<0.001	6.03 (2.06–17.64)	0.001
Molecular subtype (TNBC vs. Luminal A) ^j^	5.07 (1.29–19.87)	0.020	5.15 (1.16–22.81)	0.031
Molecular subtype (Luminal B vs. Luminal A) ^j^	1.85 (0.41–8.36)	0.423	Not included ^f^	—
ER (negative vs. positive)	8.13 (3.65–18.13)	<0.001	Not included ^g^	—
PR (negative vs. positive)	3.49 (1.60–7.58)	0.002	Not included ^g^	—
HER2 (positive vs. negative)	4.64 (2.16–9.93)	<0.001	Not included ^g^	—
ADC MIN (×10^−3^ mm^2^/s, every 100-unit reduction)	1.52 (1.24–1.87)	<0.001	1.41 (1.14–1.74)	0.002
ADC MAX (×10^−3^ mm^2^/s, every 100-unit reduction)	1.48 (1.21–1.82)	<0.001	Not included ^f2^	—
Tumor size (every 10 mm reduction)	1.42 (1.19–1.69)	<0.001	1.28 (1.05–1.56)	0.016
Ki-67 >20% (vs. ≤20%)	2.78 (1.31–5.91)	0.008	Not included	—
Contrast speed (fast vs. slow)	1.70 (0.81–3.57)	0.161	Not included ^f^	—
NME (present vs. absent)	2.44 (0.75–7.91)	0.138	Not included ^f^	—

^e^ Complete separation observed; odds ratio is incalculable; variable excluded from multivariate model. ^f^ Excluded from multivariate model due to univariate *p* > 0.10. ^f2^ ADC MAX demonstrated univariate significance (*p* < 0.001) but was removed from the multivariate model due to high collinearity with ADC MIN (r = 0.548, *p* < 0.001). ^g^ Removed from multivariate model due to strong collinearity with molecular subtype via ER/PR/HER2 status (r > 0.80). ^h^ Tumor margin Fisher exact test *p* = 0.005; logistic regression univariate *p* = 0.183; the discrepancy reflects inherent differences between the two statistical approaches. ^j^ Polytomous logistic regression model; reference category = Luminal A; model fit assessed by Hosmer–Lemeshow test (*p* > 0.05). No correction for multiple comparisons was applied. OR: odds ratio; CI: confidence interval; ADC: apparent diffusion coefficient; pCR: pathological complete response; NME: non-mass enhancement; TNBC: triple-negative breast cancer.

**Table 4:** ROC Analyses: Pre-treatment ADC and Post-treatment ADC + ΔADC.

Analysis Group	Parameters	AUC (95% CI)	Youden Cut-Off	Sensitivity (%)	Specificity (%)
PRE-TREATMENT—pCR prediction					
The entire cohort	ADC MIN	0.724 (0.641–0.807)	≤821 × 10^−3^ mm^2^/s	75.7	68.2
The entire cohort	ADC MAX	0.751 (0.671–0.831)	≤952 × 10^−3^ mm^2^/s	78.4	68.9
Luminal (A + B) ^m^	ADC MIN	0.582 (0.451–0.713)	≤891 × 10^−3^ mm^2^/s	62.5	54.7
HER2-enriched	ADC MIN	0.812 (0.703–0.921)	≤798 × 10^−3^ mm^2^/s	83.3	72.1
TNBC ^n^	ADC MIN	0.771 (0.561–0.982)	≤751 × 10^−3^ mm^2^/s	80.0	71.4
POST-TREATMENT—Residual tumor detection					
The entire cohort	Post-ADC	0.967 (0.936–0.993)	>1513 × 10^−3^ mm^2^/s	100.0	96.7
The entire cohort	ΔADC (%)	0.912 (0.862–0.955)	>85%	91.9	84.8

^n^ *n* = 19 in the TNBC subgroup (pCR(+) = 5, pCR(−) = 14); due to the low number of cases, the AUC value should be interpreted with caution. The wide confidence interval (0.561–0.982) substantially limits the reliability of this estimate; results should not be generalized beyond this cohort. ^m^ Luminal A and Luminal B cases were combined to provide a sufficient subgroup size. 95% CI calculated with the bootstrap method (1000 iterations). ΔADC (%) = [(Post-ADC − Pre-ADC MIN)/Pre-ADC MIN] × 100. Post-ADC and ΔADC AUC values were compared with the DeLong test; no statistically significant difference was found (*p* = 0.156). Potential overfitting cannot be excluded; external validation is required.

**Table 5:** Post-treatment MRI Accuracy and Post-ADC/ΔADC Analysis.

Variable	pCR(−) (*n* = 151)	pCR(+) (*n* = 37)	*p*-Value	Pathology: pCR(+)	Pathology: pCR(−)	Total
Post-ADC (×10^−3^ mm^2^/s) ^a^	1115.0 (1081.0–1216.0)	1712.0 (1703.0–1770.0)	<0.001	—	—	—
ΔADC (%) ^a^	30.5 (19.2–52.1)	138.2 (94.1–153.4)	<0.001	—	—	—
Residual tumor size (mm) ^ab^	16.0 (12.0–21.0)	— ^b^	— ^b^	—	—	—
MRI: Complete response	—	—	—	37 (TP)	21 (FP)	58
MRI: Partial/Stable	—	—	—	0 (FN)	130 (TN)	130
Total	—	—	—	37	151	188

^a^ Median (Q1–Q3); Mann–Whitney U test. ^b^ There is no residual tumor in the pCR(+) group by definition. TP: True Positive; FP: False Positive; FN: False Negative; TN: True Negative; pCR: pathological complete response; ADC: apparent diffusion coefficient; ΔADC: percentage of ADC change before and after treatment; MRI: magnetic resonance imaging.

## Data Availability

Data used in this study can be provided upon reasonable request.

## Abbreviations

The following abbreviations are used in this manuscript:

ACR	American College of Radiology
ADC	Apparent diffusion coefficient
AUC	Area under the curve
BI-RADS	Breast Imaging Reporting and Data System
CI	Confidence interval
DCIS	Ductal carcinoma in situ
DCE-MRI	Dynamic contrast-enhanced magnetic resonance imaging
DWI	Diffusion-weighted imaging
ER	Estrogen receptor
FN	False negative
FP	False positive
HER2	Human epidermal growth factor receptor 2
HR	Hormone receptor
MRI	Magnetic resonance imaging
NAC	Neoadjuvant chemotherapy
NME	Non-mass enhancement
NPV	Negative predictive value
OR	Odds ratio
pCR	Pathologic complete response
PPV	Positive predictive value
PR	Progesterone receptor
rCR	Radiologic complete response
ROC	Receiver operating characteristic
ROI	Region of interest
TIC	Time-intensity curve
TN	True negative
TNBC	Triple-negative breast cancer
TP	True positive
T2W	T2-weighted
ΔADC	Percentage change in apparent diffusion coefficient
